# The complete mitochondrial genome and phylogenetic analysis of *Cancer pagurus* (Decapoda, Cancridae)

**DOI:** 10.1080/23802359.2019.1689859

**Published:** 2019-11-13

**Authors:** Sigang Fan, Chao Zhao, Pengfei Wang, Lulu Yan, Lihua Qiu

**Affiliations:** a Key Laboratory of Aquatic Product Processing, Key Laboratory of South China Sea Fishery Resources Exploitation & Utilization, Ministry of Agriculture, South China Sea Fisheries Research Institute, Chinese Academy of Fishery Sciences, Guangzhou, PR China;; b Key Laboratory of Aquatic Genomics, Ministry of Agriculture, PR China

**Keywords:** *Cancer pagurus*, mitochondrial genome, phylogeny

## Abstract

The complete mitochondrial genome of *Cancer pagurus* was obtained using next-generation sequencing. The circular genome was 42,736 bp in length, consisting of 13 protein-coding genes, 26 transfer RNA genes, and 2 ribosomal RNA genes. The control region was not found in mitochondrial genome. Of the 41 genes, 21 were encoded by the heavy strand, while the others were encoded by the light strand. The genome composition with A + T bias (74.10%). The phylogenetic analysis suggested that *C. pagurus* was closest to *Austinograea alayseae*. The newly described mitochondrial genome may provide valuable data for phylogenetic analysis for Cancridae.

Edible crab, *Cancer pagurus*, mainly distributed along Northeast Atlantic coasts, from Morocco in the south to the northern part of Norway (FAO 2015). *Cancer pagurus* inhabits in the benthic zone and mainly hunts for molluscks (Ma et al. 2019). It is one of the most important crustacean consumed in Southern European countries (Barrento et al. 2008; EuroStat 2010). Approximately 50,000t were caught in Europe (FAO 2015). Mitochondrial DNA is usually considered as an ideal marker for population genetic research due to its maternal inheritance and fast mutation rate (Avise et al. 1987). The mitochondrial genome is an effective tool for species identification, molecular taxonomy, and population genetic analyses (Galtier et al. 2009). Therefore, the complete mitochondrial genome of *C. pagurus* and its phylogenetic relationships within crab were investigated in this study. We expect that the genomic data will provide essential information to genetic resources conservation and systematic study of *C*. *pagurus*.

Specimens of *C. pagurus* were collected from Huangsha aquatic products market in Guangzhou (23°07′N, 113°5′8″E), Guangdong province, China, and kept in the South China Sea Fisheries Institute (Guangzhou, China). Muscle was sampled and frozen in liquid nitrogen and stored at −80 °C. After sampling, the specimen was stored in 90% ethanol and deposited at the South China Sea Fisheries Research Institute Museum (Acc. Number CPGZ20190725). The mitochondrial DNA (mtDNA) was isolated by Mitochondrial DNA Isolation Kit (Haling Biotech Shanghai, Co., Ltd., Shanghai, China) and sequenced using the Illumina Hiseq Sequencing System (Illumina Inc., San Diego, CA). The clean data were acquired and assembled by the SPAdes and PRICE (Bankevich et al. 2012). The mitogenome was annotated by UGENE ORFs finder and tRNAscan-SE (http://www.cbs.dtu.dk/services/RNAmmer/). The mitogenome was submitted into GenBank database under the accession number of MN334534.

In general, the complete mitochondrial genome of crab was 1.5–1.7 kb in length (Ma et al. 2015). However, the complete mitogenome sequence of *C. pagurus* was 42,736 bp in length, which was longer than another crab’s. The overall base composition of *C. pagurus* mitogenome sequence is A: 36.3%, T: 37.8%, C: 20.1%, and G: 5.8%. The genome contained 13 protein-coding genes, 26 transfer RNA genes, and 2 ribosomal RNA genes. However, the control region was not predicted successfully in mitochondrial DNA sequence. Among 26 transfer RNA genes, there are 5 TrnLs and 3 TrnFs. Of the 41 genes, 21 were encoded by the heavy strand, and the others were encoded by the light strand. Eight protein-coding genes (*ND4*, *ND4L*, *ND5*, *ND6*, *ND2*, *COX2*, *ATP8*, and *COX3*) were initiated by ATG. Two genes (*Cox1* and *Cytb*) were started by ATA. ND1 and ATP6 were started by GTG. ND3 was initiated by ATC. Ten PCGs (*ND2*, *ND3*, *ND6*, *COX3*, *ATP6*, *Cytb*, *ND4*, *ND4L*, *ND1*, and *ATP8*) terminate with the typical TAA or TAG as stop codon, while three PCGs (*Cox1*, *Cox2*, and *ND5*) end with T––. Twenty-six tRNA genes ranged in size from 55 to 75 bp.

The phylogenetic tree was constructed based on 13 concatenated protein-coding genes from 14 crab species from the Genbank database, by maximum likelihood (ML) method. *Hapiosquilla harpax* was used as an outgroup for tree rooting (Figure 1). It was demonstrated that *C. pagurus* was clustered with *A. alayseae*, which may suggest Cancroidea was close with Bythograeidae. In all, this genome will contribute to future phylogenetic studies of Cancridae and population genetic analyses for *C. pagurus.*

**Figure 1.**
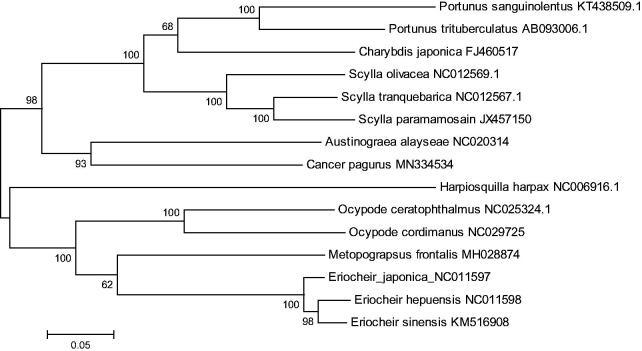
Phylogenetic tree of *C. pagurus* and related species based on maximum likelihood (ML) method with *Harpiosquilla harpax* as an outgroup.
